# Ubiquitous expression of the rtTA2S-M2 inducible system in transgenic mice driven by the human hnRNPA2B1/CBX3 CpG island

**DOI:** 10.1186/1471-213X-7-108

**Published:** 2007-09-27

**Authors:** Eleni Z Katsantoni, Nora E Anghelescu, Robbert Rottier, Matthijs Moerland, Michael Antoniou, Rini de Crom, Frank Grosveld, John Strouboulis

**Affiliations:** 1 Department of Cell Biology, Erasmus University Medical Center, PO Box 2040, 3000 CA Rotterdam, The Netherlands; 2 Hematology Division, Biomedical Research Foundation, Academy of Athens, 4 Soranou Ephesiou, 115 27 Athens, Greece; 3 Nuclear Biology Group, Division of Medical and Molecular Genetics, GKT School of Medicine, King's College London, Guy's Hospital, London SE1 9RT, UK; 4 Institute of Molecular Oncology, BSRC "Alexander Fleming", PO Box 74145, 166 02 Varkiza, Greece; 5 Gene Controls Mechanism and Disease, MRC Clinical Sciences Centre, Faculty of Medicine, Imperial College London, Hammersmith Hospital Campus, Du Cane Road, London W12 0NN, UK

## Abstract

**Background:**

A sensitive, ubiquitously expressed tetracycline inducible system would be a valuable tool in mouse transgenesis. However, this has been difficult to obtain due to position effects observed at different chromosomal sites of transgene integration, which negatively affect expression in many tissues. The aim of this study was to test the utility of a mammalian methylation-free CpG island to drive ubiquitous expression of the sensitive doxycycline (Dox) inducible rtTA2S-M2 Tet-transactivator in transgenic mice.

**Results:**

An 8 kb genomic fragment from the methylation-free CpG island of the human hnRNPA2B1-CBX3 housekeeping gene locus was tested. In a number of transgenic mouse lines obtained, rtTA2S-M2 expression was detected in many tissues examined. Characterisation of the highest expressing rtTA2S-M2 transgenic mouse line demonstrated Dox-inducible GFP transgene expression in many tissues. Using this line we also show highly sensitive quantitative induction with low doses of Dox of an assayable plasma protein transgene under the control of a Tet Responsive Element (TRE). The utility of this rtTA2S-M2 line for inducible expression in mouse embryos was also demonstrated using a GATA-6 Tet-inducible transgene to show specific phenotypes in the embryonic lung, as well as broader effects resulting from the inducible widespread overexpression of the transgene.

**Conclusion:**

The ubiquitously expressing rtTA2S-M2 transgenic mouse line described here provides a very useful tool for studying the effects of the widespread, inducible overexpression of genes during embryonic development and in adult mice.

## Background

Controlling gene expression in a temporally and spatially inducible manner is an important aspect of transgenic approaches. A key development has been the application of gene expression systems based on the tetracycline-resistance (tet) operon of the Tn10 transposon of E. coli. In the absence of tetracycline, the tet repressor (tetR) DNA binding protein binds to a defined DNA operator sequence (tetO) and suppresses transcription. Addition of tetracycline (Tc) causes a conformational change in TetR preventing it from binding to tetO [1]. In the tet transactivator (tTA) system, tetR was fused to VP16, a strong transcriptional activator, so that it now activates expression upon binding to TetO in the absence of Tc [1]. It was further improved by developing a reverse tet transactivator (rtTA) which requires Tc for binding tetO for transactivation, thus eliminating long-term exposure to Tc [2]. Also, doxycycline (Dox) was introduced as an inducer due to its superior qualities of lower toxicity, longer half-life and high bioavailabilty. Finally, the rtTA2S-M2 variant generated by mutagenesis constitutes a significant improvement over the original rtTA since it is induced at a 10-fold lower Dox concentration compared to rtTA, it shows no background DNA binding and it is also more stable in mammalian cells [3].

Application of the rtTA system in mice requires two transgenic lines: one line expressing the rtTA under an appropriate promoter and a second line carrying the target transgene of interest linked to a Tet-responsive element (TRE) containing tetO sequences. However, the efficiency of the tet system in transgenic animals is often negatively impacted by the chromosomal site of integration of the transgenes. This becomes particularly important when ubiquitous induction in several tissues is desirable. Whereas chromosomal position effects can be partly alleviated by the use of gene domain regulatory elements such as Locus Control Regions or insulators [4], these elements are often tissue specific. By contrast, regulatory elements associated with ubiquitously expressed housekeeping genes are active in all tissues. For example, methylation-free CpG islands associated with the 5' ends of housekeeping genes are known to be localized in regions of active chromatin [5]. Previous evidence from cell transfection experiments has suggested that transgenes containing CpG islands have the potential to protect from position effects, even when integrated in heterochromatin. For example, large fragments spanning CpG islands from the ubiquitously expressed human TBP-PSMB1 and hnRNPA2B1-CBX3 loci were able to protect from heterochromatic silencing [6,7]. These regions are structurally similar in that they contain divergently transcribed promoters embedded within an extended CpG island. In the hnRNPA2B1-CBX3 locus the two divergently expressed genes code for heterogeneous ribonucleoprotein A2/B1 and for chromobox homolog 3 [8]. Assays in mammalian cells showed that the hnRNPA2B1-CBX3 CpG island gave rise to reproducible, stable and non-variegated expression from the endogenous hnRNPA2B1 promoter, even when integrated in centromeric heterochromatin [6]. Furthermore, the coupling of the hnRNPA2B1-CBX3 CpG island to the CMV promoter gave substantial improvements in the level and stability of expression, resulting in improved production of recombinant proteins in CHO cells [9]. These observations suggest that methylation-free CpG islands may harbor dominant chromatin remodeling functions. In this report, we show that the hnRNPA2B1-CBX3 CpG island is sufficient to generate an efficient, ubiquitously expressed rtTA inducible system in transgenic mice.

## Results and discussion

**Figure 1:**
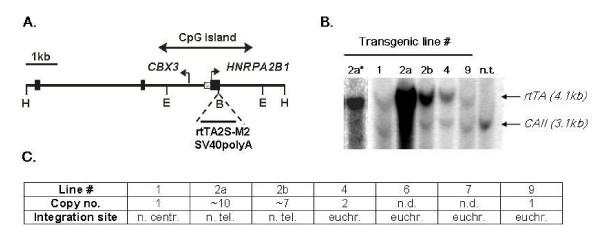
(A) Diagram of the 8 kb Hind III (H) genomic fragment showing the hnRNPA2B1 and CBX3 promoters and the CpG island that spans them. The Bgl II (B) site used for cloning the rtTA2S-M2SV40polyA cDNA is shown. The EcoRI (E) sites used for determining transgene copy numbers are also shown. (B) Southern blot of EcoRI digests of genomic DNA from the hnRNPA2B1-rtTA2S-M2 transgenic mouse lines to determine transgene copy numbers. Probes used: an rtTA2S-M2 fragment detecting a 4.1 kb band and, as control, a CAII probe detecting a 3.1 kb band from the endogenous carbonic anhydrase II locus. Lane 2a* shows a different exposure (n.t.: non transgenic). (C) Summary table of the transgene copy numbers for the different lines and of the chromosomal integration sites as determined by FISH (n.d.: not determined; n.centr.: near centromere; n.tel.: near telomere).

We used an ~8 kb DNA fragment encompassing the human hnRNPA2/B1-CBX3 gene locus CpG island, which includes the two divergently transcribed promoters (Fig. 1A). The rtTA2S-M2 variant was cloned under the control of the hnRNPA2/B1 promoter as shown (Fig. 1A) and the entire 8 kb fragment was purified and microinjected into mouse fertilized eggs. A total of 10 founders transgenic for the hnRNPA2B1-CBX3/rtTA2S-M2 construct were generated and seven transgenic lines were established through transmission of the transgene. Transgenic founder 2 had two integration sites, which separated upon breeding to generate lines 2a and 2b. Integrity of the transgenes was established by Southern blotting using the 8 kb DNA microinjection fragment as probe (not shown). For the majority of transgenic lines, we also determined transgene copy numbers (Fig. 1B) and chromosomal integration sites using FISH (summarized in Fig. 1C).

**Figure 2:**
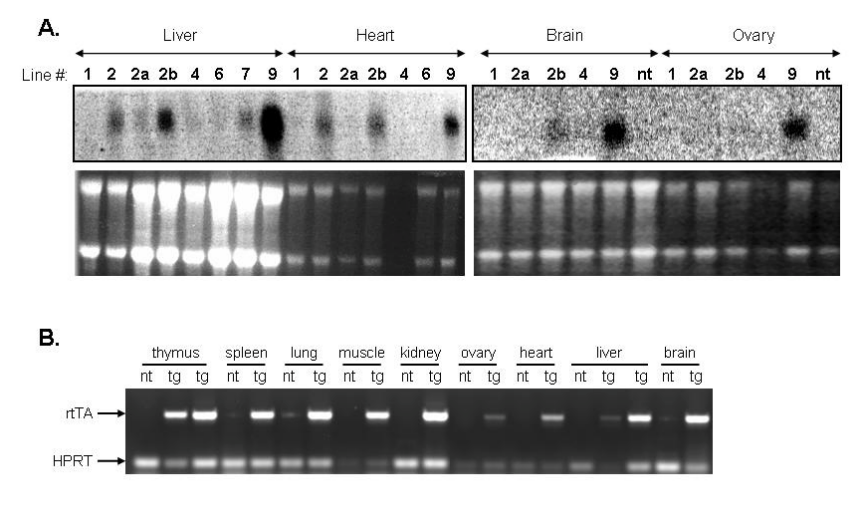
(A) Northern blot analysis of rtTA2S-M2 transgene expression in different tissues in transgenic lines. Probe used was the rtTA2S-M2 fragment. Lower panels: rRNA loading control visualized by ethidium bromide staining. (B) RT-PCR of rtTA2S-M2 transgene expression in different tissues in transgenic line 9 (nt: non transgenic, tg: transgenic). Expression of the endogenous HPRT gene was used as control.

We next carried out Northern blot analysis to determine the tissue distribution and relative expression levels of the rtTA2S-M2 transgene in the different lines. Line 9 is clearly the highest expressing line in all tissues tested, lines 2a and 2b expressed weakly in some tissues, whereas lines 1, 4, 6 and 7 barely expressed (Fig. 2A). These data show that transgene expression levels are not copy number dependent and are prone to position effects. This was confirmed by quantitative RT-PCR for lines 1, 2a, 2b, 4 and 9 in heart and liver RNA. Plotting normalized levels of expression against transgene copy numbers shows no direct correlation between expression levels and copy numbers, for example, the single copy line 9 expresses at much higher levels than the multi copy line 2a (Additional file 1). The chromosomal sites of integration of the transgenes cannot readily account for these results. For example, line 4 carrying 2 copies of the transgene in a euchromatic site does not express, whereas single copy line 9, also integrated in euchromatin, expresses at high levels. These observations contrast with previous work in transfected cells showing that a broader 16 kb genomic fragment spanning the hnRNPA2B1-CBX3 locus expressed consistently even when integrated in centromeric heterochromatin [6], or that the 8 kb hnRNPA2B1-CBX3 fragment when linked to a CMV promoter showed consistently detectable, though variable, transgene expression for all integration events [9]. Reasons that could account for these differences include the requirement of additional elements for position independent transgene expression which are lacking from the 8 kb fragment we used [6], or that the use of antibiotic resistance in cell transfections introduces a bias for open chromatin integration, particularly when using the CMV promoter [9].

**Figure 3:**
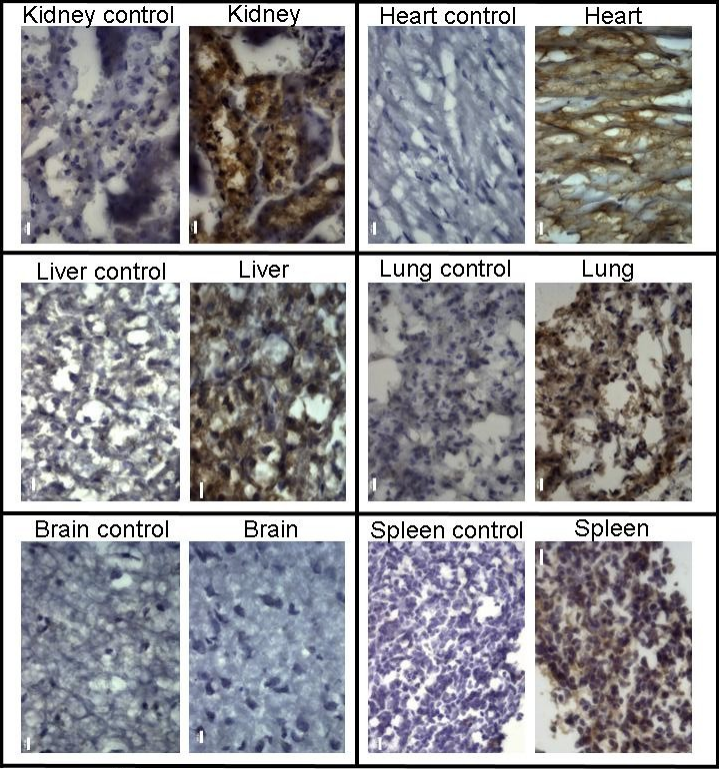
Immunohistochemistry on sections from the heart, lung, spleen, kidney, liver and brain of hnRNPA2B1-rtTA2S-M2/TRE-GFP double transgenic mice (top panels) and of control TRE-GFP mice (lower panels), all treated with 2 mg/ml Dox for 3–4 days and stained with an anti-GFP antibody (scale bars: 10 μm).

We next focused on the utility of the highly expressing line 9 as a tool for widespread inducible transgene expression upon Dox treatment. We first obtained further evidence that the rtTA2S-M2 transgene is indeed widely expressed in this line. By employing RT-PCR, we detected expression of the transgene in all tissues assayed (Fig. 2B), thus confirming the ubiquitous expression of the rtTA2S-M2 transgene driven by the hnRNPA2B1-CBX3 CpG island. We next generated three transgenic lines carrying a TRE-GFP reporter construct and crossed them with line 9. Analysis of GFP expression in the liver of adult mice treated with Dox showed that TRE-GFP line 2 was induced more efficiently than TRE-GFP lines 1 and 3 (data not shown). Thus, TRE-GFP line 2 was used in all subsequent experiments. Because of the high levels of autofluorescence observed in the liver and other tissues (not shown), we assayed induction of GFP expression in different tissues by immunohistochemistry with an anti-GFP antibody (Fig. 3). We observed clear induction of GFP expression in the kidney, lung, heart, spleen and liver specifically upon Dox treatment of double transgenic mice (Fig. 3). Thus, taken together these results show that the rtTA2S-M2 transgene is expressed in the liver, heart, brain, ovary, kidney, lung, spleen, muscle, thymus and blood (see below) with Dox inducibility demonstrated in the liver, kidney, heart, lung, spleen and blood (see below). To-date there have been few other studies reporting the use of the improved rtTA2S-M2 variant in transgenic mice, for example, for the tissue specific inducible expression of genes in the retinal ganglia cells or the brain [10,11]. Transgenic mouse line 9 represents an important addition to the transgenic tools that are available exploiting the superior properties of the rtTA2S-M2 system.

Despite robust expression of the rtTA2S-M2 transgene in the brain of line 9 (Fig. 2A and 2B), we were unable to detect GFP induction in the brain (Fig. 3), even after treatment with 2 mg/ml Dox for up to 18 days (not shown). This is most likely due to the well-known limited penetration of the blood-brain barrier by Dox. The study by Michalon et al. [10] has suggested that induction in the brain may require treating mice for longer periods with higher doses of Dox. However, in the case of line 9 where the rtTA2S-M2 transgene is expressed in many tissues, one needs to keep in mind the potential side effects that prolonged treatment with high amounts of Dox may have in tissues other than the brain [12].

**Figure 4:**
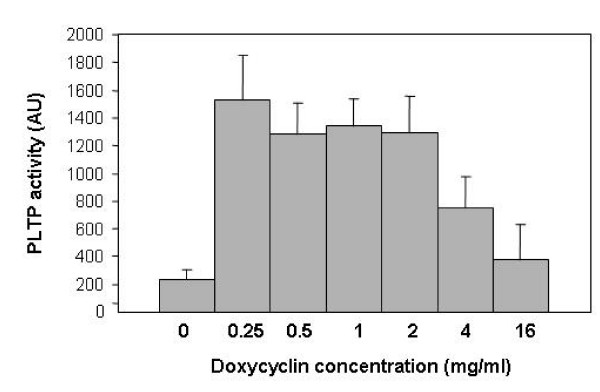
PLTP activity in serum following administration of increasing amounts of Dox in the drinking water, as indicated. PLTP activity is expressed as percentage of human reference plasma.

In order to test the dose response of line 9 to Dox, we crossed it with a transgenic mouse line expressing the human phospholipid transfer protein (PLTP) under the control of a Tet responsive element (TRE), which was created for the purposes of another study (MM and RdC). PLTP is a plasma protein with several key roles in lipoprotein metabolism and, potentially, atherogenesis [13,14]. We used a sensitive assay to measure PLTP activity in the serum of double line 9/TRE-PLTP transgenic mice as a means of quantifying induction with increasing Dox concentrations added to the drinking water. We found a 6- to 7-fold induction in PLTP activity for Dox concentrations ranging from 0.25 to 2.0 mg/ml (Fig. 4). PLTP activity appeared to decline with increasing Dox dosage (4.0 to 16.0 mg/ml, Fig. 4), however, these concentrations of Dox are much higher than those normally used in mice for induction. Thus, taken together, the data presented in Figure 4 establish a useful range of Dox concentrations for inducing PLTP activity and, in addition, demonstrate the superior sensitivity of induction in the serum of the rtTA2S-M2 variant in transgenic mice with as little as 0.25 mg/ml of Dox.

**Figure 5:**
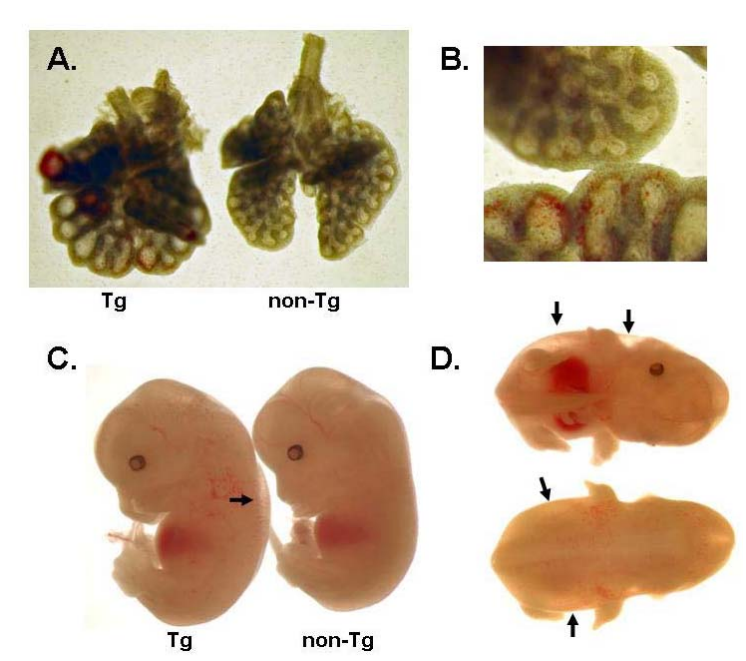
(A-B) Whole mount view of the lungs of the double transgenic and the control littermate showing the enlargement of the airspaces, which are highlighted in B (top is the non-Tg and lower is the Tg lung). (C) Lateral views of a double transgenic hnRNPA2B1-rtTA2S-M2 line 9/TRE-myc-GATA-6 (Tg) and a control litter mate (non-Tg) at gestational age 12.5 dpc 24 hours after start of induction with Dox. The arrow indicates the clearly visible oedema after induction of the GATA-6 transgene. (D) Ventral (top) and dorsal (bottom) views of the double transgenic hnRNPA2B1-rtTA2S-M2 line 9/TRE-myc-GATA-6 to show the clear indication of oedema (arrows).

In order to demonstrate the utility of line 9 for Dox inducible expression in mouse embryos, we established a sensitive genetic system based on a myc-tagged murine GATA-6 transgene under the control of a TRE. GATA-6 is an essential transcription factor with important functions in endoderm differentiation (review, [15]). Importantly, GATA-6 overexpression in transgenic embryos results in branching defects in the lung, showing that lung development is sensitive to GATA-6 dosage [16]. Microinjection of the TRE-myc-GATA-6 fragment into fertilized eggs resulted in 30 live born pups, of which 9 carried the transgene as deduced by Southern blot analysis (not shown). Three lines were chosen for further analysis based on the intensity of the signal on Southern blots. All heterozygous and homozygous TRE-myc-GATA-6 mice were viable and showed no defects. Timed pregnancies were set up between HNRPA2B1-rtTA2S-M2 homozygous mice and TRE-myc-GATA-6 heterozygous mice. Dox was given to pregnant dams for one day before isolating embryos (see Methods). Since it has been previously shown that overexpression of the myc-GATA-6 transgene results in defects in the differentiation of the airway epithelium of the lung [16], we examined the lungs of double transgenic pups that were treated with Dox. We found striking similarities with the published lung phenotype, in that the airways appear dilated with some bleeding also evident (Fig. 5A and 5B). The lung phenotype was observed in all three independent TRE-myc-GATA-6 lines and appeared only in double transgenics treated with Dox. There was no obvious lung phenotype in double transgenics not treated with Dox (not shown). Furthermore, Dox treatment of pregnant dams for only one day also resulted in gross morphology defects in that the embryos showed loosening of the skin and signs of oedema (Fig. 5C and 5D). These observations could be due to interference of normal GATA factor functions in the skin as a result of induced myc-GATA-6 overexpression [17,18]. Induction of the transgene for more than one day resulted in a general growth arrest and death in utero. Thus, these observations reveal defects that are specific for the induction of the myc-GATA-6 transgene and demonstrate the utility of hnRNPA2B1-rtTA2S-M2 line 9 in studying the effects of the widespread inducible overexpression of specific factors in embryonic development. Alternatively, this system could be used for timed rescue experiments of mice that have essential genes knocked out.

## Conclusion

In conclusion, we showed that the methylation free CpG island of the human housekeeping hnRNPA2B1-CBX3 gene locus is capable of driving expression of a linked rtTA2S-M2 transgene in mice in all tissues that were examined. Transgene expression was not found to be copy number related, suggesting that the CpG island does not completely escape chromosomal position effects in vivo. However, in line 9 where the transgene is most active, it is expressed in all tissues tested. Furthermore, using this system we demonstrated that the highly expressing line 9 is a very useful tool for the inducible, sensitive expression of transgenes in a variety of tissues in response to Dox treatment. Importantly, the inducible transgenic system reported here can be used to examine the effects of inducing widespread overexpression of transcription factors during mouse development.

## Methods

### DNA constructs

An 8 kb genomic fragment spanning the CpG island of the HNRPA2B1-CBX3 locus was used to express the rtTA derivative rtTA2S-M2. The latter was obtained as an EcoRI-HindIII fragment, including the SV40 polyA sequence, from plasmid pUHrT62-1 [3] and was blunt-end cloned into a Bgl II site in the first exon of the HNRPA2B1 gene. The cDNAs for GFP and human PLTP [19] and murine N-terminally myc-tagged GATA-6 [20] were cloned under the control of a TetO-CMV promoter in plasmids pTRE (GFP and PLTP) or pTRE-Tight (GATA-6) (Clontech).

### Transgenic mice

HNRPA2B1/rtTA2S-M2 was released from vector sequences as an 8 kb HindIII fragment (Fig. 1A). TRE-GFP-SV40polyA and TRE-hPLTP-SV40polyA were released from vector sequences as XhoI/PvuII fragments. TRE-myc tag-GATA-6-SV40polyA was released as an XhoI fragment. All fragments were purified by salt gradient centrifugation, as previously described [21], microinjected at approximately 0.5 ng/μL into the pronucleus of fertilized eggs of FVB/N mice and transplanted into the oviducts of pseudopregnant B10xCBA mice [22]. Transgenic founders were identified by Southern blotting using the rtTA2S-M2, GFP, hPLTP and GATA-6 fragments as probes. The integrity of the HNRPA2B1/rtTA2S-M2 transgene was checked by hybridization using the entire HNRPA2B1/rtTA2S-M2 construct as probe. Transgene copy numbers for the HNRPA2B1/rtTA2S-M2 lines were determined from the intensity of fragments using an EcoRI/BamHI rtTA2S-M2 probe, together with a 0.9-kb Pvu I probe detecting the endogenous mouse carbonic anhydrase II (CA-II) gene. PhosphorImager analysis was performed using ImageQuant software (Molecular Dynamics, Sunnyvale, CA). All animal experiments described in this work conformed to national and institutional guidelines.

### DNA fluorescence in situ hybridization analysis

FISH was carried out to determine chromosomal sites of transgene integration, as previously described [23,24]. The specific probe used was the biotin-labeled HNRPA2B1/rtTA2SM2 injection fragment.

### Doxycycline induction

Mice (2–4 months old) were kept on drinking water containing 5% sucrose for three days, followed by drinking water containing 2 mg/ml doxycycline hydrochloride (Sigma) plus 5% sucrose for 3–4 days. Doxycycline-containing drinking water was protected from light. Negative control mice were kept on drinking water containing 5% sucrose for the same time period. The drinking water was replaced every 2–3 days. Doxycycline induction of the myc-GATA-6 transgene expression in embryos: after the identification of a vaginal plug in the morning, dams bearing single and double (HNRPA2B1/rtTA2S-M2 and myc-GATA-6) transgenic pups were given doxycycline in the drinking water (2 mg/ml supplemented with 5% sucrose) for 1–4 days, after which embryos were isolated at different gestational ages and processed for further analysis.

### Northern blot analysis

RNA was isolated using the Trizol reagent and according to the manufacturer's instructions (Invitrogen). Northern blot analysis was carried out with 5 μg of total RNA as previously described [25]. The rtTA2S-M2 fragment was used as probe for hybridization.

### RT-PCR and Quantitative RT-PCR

RNA was treated with RQ1 RNase-Free DNase I (Promega) for 30 minutes at 37°C according to the manufacturer's instructions. DNase-treated RNA samples were reverse transcribed with Superscript (Invitrogen) using oligo dT. Control reactions without reverse transcriptase (RT) were also performed. Real-time PCR reactions were performed in triplicate with a Chromo 4, MJ Research RealTime PCR Cycler, as previously described [26]. Normalization for the amount of template was done using primers specific for exon 8 of the mouse HPRT gene. Primer sequences for rtTA2S-M2 and HPRT are available upon request. Data were analyzed using the Chromo 4, MJ Research RealTime PCR software and statistical analysis was done using the comparative C_T _method for the relative quantitation of results [27]. Post amplification denaturation curves showed that the primer pairs generated single products.

### PLTP activity assay

Blood samples were collected from the orbital plexus of mice before the sucrose run-in period, after three days of plain sucrose administration and after three days of doxycycline administration. Blood was centrifuged at 2800 rpm for 15 minutes at 4°C and plasma samples stored at -80°C. PLTP activity was measured using a phospholipid vesicle-HDL system [28]. Radiolabelled phospholipid vesicles were prepared by mixing egg phosphatidylcholine (Sigma) with [^14^C]dipalmitoylphosphatidylcholine (Amersham) and butylated hydroxytoluene (Sigma), followed by drying under N2 and sonification. EDTA-plasma samples (5 μl of plasma diluted 1:150) and phospholipid vesicles were incubated in the presence of isolated human HDL for 45 minutes at 37°C. After incubation, the vesicles were precipitated and the radioactivity transferred to HDL was counted in the supernatant by liquid scintillation. PLTP activity was expressed as a percentage of human reference plasma (100% is equivalent to 14 μmol/ml/h).

### Perfusion of mice

Mice were sacrificed using an overdose of isoflurane (1-chloro-2,2,2-trifluoroethyl-difluoromethyl-ether). Subsequently, *in situ *perfusion fixation was performed by flushing 20 ml PBS through a cardiac puncture followed by 20 ml 4% (v/v) paraformaldehyde in PBS.

### Immunohistochemistry

Immunohistochemistry was performed on cryosections as previously described [29], with the following modifications: endogenous peroxidase activity was inhibited by a 30 min incubation in 1% H_2_O_2 _in methanol. A rabbit polyclonal anti-GFP antibody (Abcam ab290) was used in a dilution of 1:200. Rabbit IgG (Santa Cruz) was used as a negative control in dilution of 1:500. Anti rabbit HRP-conjugated IgG was used as secondary antibody at a 1:500 dilution. Antigen-antibody complexes were visualized by incubation in substrate solution, containing hydrogen peroxide and 3,3'-diaminobenzidine HCl (DAB substrate kit for peroxidase, Vector Laboratories). Photographs were recorded with a digital camera (Olympus DP 50) on a light Olympus Microscope (BX 40 with U-DO Dual View).

## Competing interests

The author(s) declares that there are no competing interests.

## Authors' contributions

EZK performed the experiments for the characterization of the hnRNPA2B1-rtTA2S-M2 transgenic lines, executed all the expression and immunohistochemical analyses of the hnRNPA2B1-rtTA2S-M2 line 9/TRE-GFP transgenics and wrote the manuscript. NEA made the hnRNPA2B1-rtTA2S-M2, TRE-GFP and TRE-PLTP DNA constructs, microinjected them in mouse fertilized eggs and established the transgenic mouse lines. RR generated the TRE-GATA-6 transgenic mice and performed the Tet inducible GATA-6 overexpression experiments in embryos. MM performed the Dox dosage dependent PLTP activity assay. RdC designed and supervised the PLTP project. MA carried out the initial characterization of the 8 kb hnRNPA2B1 CpG island locus construct. FG has made important conceptual contributions to the project and was involved in revising and critically correcting the manuscript. JS designed and supervised the project and wrote the manuscript. All authors read and approved the final manuscript.

## Supplementary Material

Additional file 1 suppl fig 1 katsantoni et al. GFP expression levels in liver an heart RNA as determined by RT-PCR and plotted against transgene copy numbers. Click here for file
