# Supplementary figures and images for: Ubiquitous expression of the rtTA2S-M2 inducible system in transgenic mice driven by the human hnRNPA2B1/CBX3 CpG island
Source: BMC Dev Biol. 2007 Sep 27;7:108. doi: 10.1186/1471-213X-7-108 (PMC2080639; doi:10.1186/1471-213X-7-108)

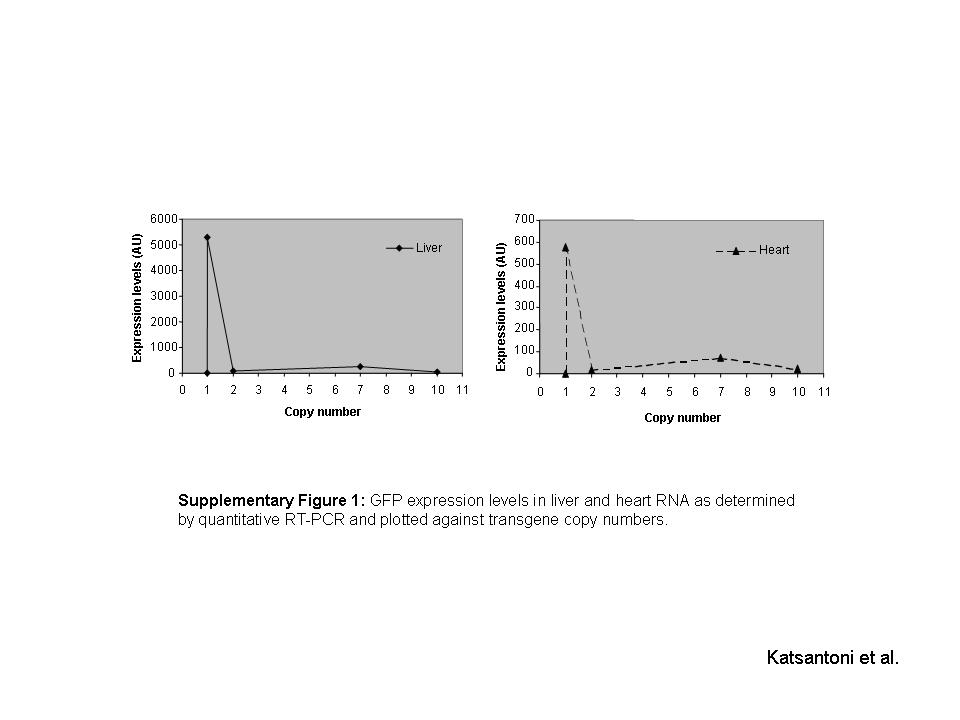

Supplement: Additional file 1 — suppl fig 1 katsantoni et al. GFP expression levels in liver an heart RNA as determined by RT-PCR and plotted against transgene copy numbers. [file 1471-213X-7-108-S1.jpeg]
